# Supplementary material for: Association between oral microbiome and seven types of cancers in East Asian population: a two-sample Mendelian randomization analysis
Source: Front Mol Biosci. 2023 Nov 21;10:1327893. doi: 10.3389/fmolb.2023.1327893 (PMC10702768; doi:10.3389/fmolb.2023.1327893)
Supplement: Supplementary file 1 [file DataSheet1.ZIP › Supplementary Materials/MR plots of saliva/saliva═╝/Prostate cancer/pheno.2592_to_prostate cancer_forest.pdf]

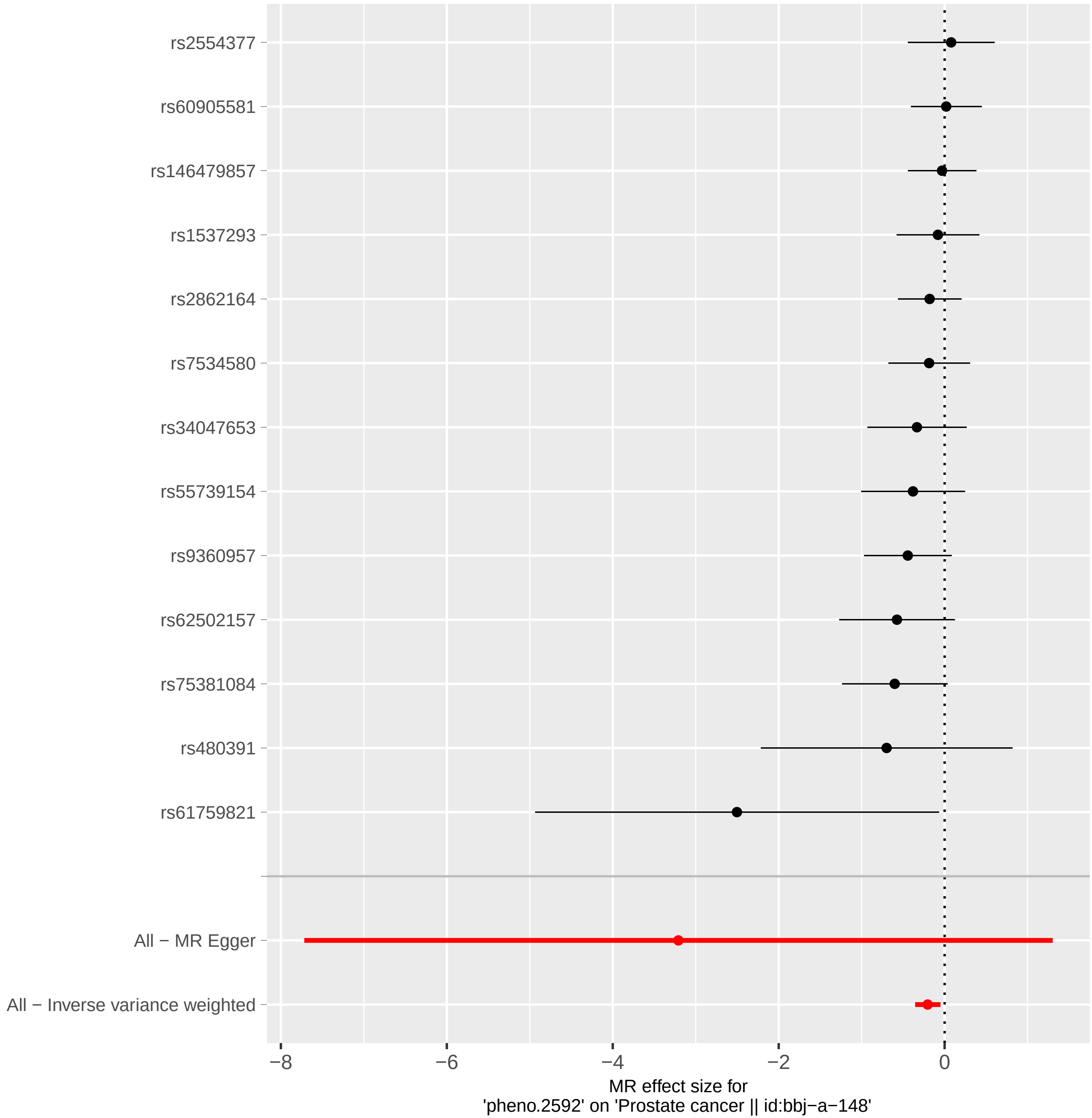
rs2554377
rs60905581
rs146479857
rs1537293
rs2862164
rs7534580
rs34047653
rs55739154
rs9360957
rs62502157
rs75381084
rs480391
rs61759821
All − MR Egger
All − Inverse variance weighted
−8
−6
−4
−2
0
MR effect size for
'pheno.2592' on 'Prostate cancer || id:bbj−a−148'
